# The English Longitudinal Study of Ageing (ELSA): Depressive symptoms and physical performance

**DOI:** 10.1186/1753-6561-7-S4-S8

**Published:** 2013-08-23

**Authors:** Panayotes Demakakos

**Affiliations:** 1 Department of Epidemiology and Public Health, University College London (UCL), London, UK

## 

The English Longitudinal Study of Ageing (ELSA) is a panel study of community-dwelling people aged 50 years or older in England. ELSA started in 2002-03 (baseline) with a sample of 12,099 people, who were recruited from households that have earlier participated in the Health Survey for England. The ELSA sample is designed to be nationally representative and thus far it consists of four cohorts that have been introduced to the study at different time points.

ELSA is a multidisciplinary study that aims to explore the dynamic relationships between health and disability, social participation, socioeconomic position, and quality of life at older ages. After the baseline, follow-up interviews take place every two years and health examinations every four years (the first health examination took place in 2004-05). Data on self-reported chronic diseases; limitations in activities of daily living and other disability; symptoms; geriatric syndromes; gait; cognition; pensions and income; wealth; housing; demographics; social participation and other psychosocial factors; well-being and quality of life; and health behaviours are collected during a personal (face-to-face) interview. Blood samples and measurements of lung function, strength and balance, and blood pressure are collected by a nurse during a health examination. Both interviews and health examinations take place at respondents’ home. More information about the study can be found at: http://www.ifs.org.uk/ELSA.

The study below is an example of the complex longitudinal analysis that could be performed using the ELSA data. We studied the association between depressive symptoms (measured using the eight-item Centre for Epidemiological Studies-Depression scale) and gait speed (m/s). Our aim was to explore whether this association is bidirectional at older ages. We used four repeated measurements of both depressive symptoms and gait speed over six years of follow-up (from 2002-03 to 2008-09). We estimated conditional models using Generalized Estimating Equations. These models were initially unadjusted and then gradually adjusted for an earlier measurement of the outcome measure, time, and covariates including socio-demographic, clinical, behavioural, psychosocial, and cognitive factors. We found that the association between depressive symptoms and gait speed was bidirectional. Slower gait speed was a predictor of concurrent and future elevated depressive symptoms and sub-threshold and elevated depressive symptoms were predictors of concurrent and future slower gait speed. These associations remained significant in the final model except for the lagged association between slower gait speed and future elevated depressive symptoms. Depressive symptoms and physical decline appear to be comorbid and mutually related at older ages.

